# Why do pregnant women prefer cesarean birth? A qualitative study in a tertiary care center in Southern Thailand

**DOI:** 10.1186/s12884-020-03525-3

**Published:** 2021-01-06

**Authors:** Chitkasaem Suwanrath, Sopen Chunuan, Phawat Matemanosak, Sutham Pinjaroen

**Affiliations:** 1 Department of Obstetrics and Gynecology, Faculty of Medicine, Prince of Songkla University, Hat Yai, Songkhla 90110 Thailand; 2 Department of Maternal-Newborn Nursing and Midwifery, Faculty of Nursing, Prince of Songkla University, Hat Yai, Songkhla 90110 Thailand

**Keywords:** Cesarean section, Fear of childbirth, Maternal request, Negative birth experience, Auspicious birth dates

## Abstract

**Background:**

Increasing worldwide rates of cesarean section are of global concern. In recent years, cesarean births upon maternal request have become a hotly debated issue. Hence, this study aimed to explore maternal reasons for cesarean preference without medical indications.

**Methods:**

A descriptive qualitative study was conducted, using in-depth interviews with 27 pregnant women who preferred cesarean birth, attending antenatal care in Songklanagarind Hospital from September 2018 to June 2019. Data were analyzed using content analysis.

**Results:**

Maternal reasons for cesarean preference were classified into six main categories: fear of childbirth, safety concerns related to health risk perceptions, negative previous birth experiences, positive attitudes toward cesarean birth, access to biased information and superstitious beliefs in auspicious birth dates. Most women had more than one reason for opting cesarean birth.

**Conclusion:**

Several reasons for cesarean birth preference have been elucidated. One striking reason was superstitious beliefs in auspicious birth dates, which are challengable for obstetricians to deal with. Obstetricians should explore the exact reasons why women request cesarean birth in order to prevent or diminish unnecessary cesarean births.

**Supplementary Information:**

The online version contains supplementary material available at 10.1186/s12884-020-03525-3.

## Background

Cesarean section is a lifesaving procedure for mothers and newborns in certain circumstances. It can, however, lead to adverse maternal and fetal outcomes [1, 2]. Currently, cesarean section rates have been rising globally. Cesarean section rates in Thailand have been significantly increasing during the past three decades from 15.2% in 1990 to 32.5% in 2017 [3, 4]. Factors contributing to this increase include delayed childbearing, policies promoting repeat cesarean section, refusal to offer vaginal birth after cesarean section, wide use of continuous electronic fetal monitoring, use of epidural analgesia, fear of malpractice liability, professional practice style, professional expectations for work-life balance, reimbursement systems, financial incentives, maternal request and lack of regulations [5–8].

Cesarean section upon maternal request is a hotly debated issue as one of the contributing factors to increased cesarean section rates [6–8]. These rates have been estimated to be 4–18% [2]. The Royal Thai College of Obstetricians and Gynecologists just announced a position statement against cesarean sections without medical indication, in order to control cesarean section rates [9]. Our institution, Songklanagarind Hospital, a referral center in Southern Thailand, does not allow cesarean sections to be performed without medical indications. Based on the database, however, from our Medical Statistics Unit, cesarean section rates have risen from 35% in 1999 to 58% in 2019.

Previous qualitative studies in other countries revealed that factors associated with cesarean preference were fear of pain, previous cesarean birth, previous negative birth experiences, multiparity, low education level and higher maternal age [10–12]. A recent systematic review of 65 studies indicated six main reasons related to mode of birth preference: 1) maternal perceptions of safety; 2) fear of pain; 3) previous birth experiences; 4) encouragement or dissuasion from health professionals; 5) social and cultural influences; and 6) access to information and educational levels [13]. It is of interest to explore reasons why some women preferred cesarean birth. Such studies are limited in our country, where cesarean section rates continue to rise. Therefore, we conducted this qualitative study to explore reasons why women preferred cesarean birth in Songklanagarind Hospital.

## Methods

This qualitative study was conducted under the research project “Knowledge and attitudes of pregnant women regarding modes of birth”, registered with the Institutional Review Board of the Faculty of Medicine, Prince of Songkla University (REC.61–177–12-4). Written informed consent was obtained from all participants before entry into the study. Participants were purposefully selected from those in the above-mentioned project, who attended antenatal care in Songklanagarind Hospital from September 2018 to June 2019 and indicated that cesarean section was their preferential mode of birth. They were invited to participate and assured with regard to confidentiality and anonymity. Inclusion criteria of the main project were: 1) singleton pregnancy; 2) gestational age ≥ 20 weeks; and 3) no maternal or fetal indications for cesarean section. Exclusion criteria were women who could not read or write or understand Thai language or whose fetuses had obvious anomalies.

In-depth interviews were performed to explore the reasons why pregnant women preferred having cesarean birth. Women were individually interviewed by the third author (PM) in the antenatal clinic in a private room using a narrative style, opening with: “Could you please tell me the reasons why you choose to have cesarean birth?” If the concept was not clear, participants were asked to explain in more detail, until the reasons were clarified. The interview guide is available in the supplementary file. Each interview lasted 20–30 min. All conversations were audio-recorded and transcribed verbatim by the interviewer. The material was evaluated by the first (CS) and second authors (SC), as being saturated after interviewing 27 women with sufficient confidence to answer the research question.

### Data analysis

Data were analyzed by the first (CS) and second authors (SC) independently, using content analysis including four stages: 1) decontextualization, 2) recontextualization, 3) categorization and 4) compilation [14]. Each author read through the transcripts and identified meaning units in the text, condensed meaning units and then labeled each unit with a code. Codes were inductively generated and themes and categories identified. Each stage was performed and revised several times in order to verify the information. Results were then discussed to obtain consensus. Transcripts were discussed using an editing analysis style and drafting categories based upon the data. Finally, synthesis of the condensates into re-conceptualized descriptions was performed and approved by all authors.

## Results

A total of 27 pregnant women underwent in-depth interviews. Age ranged from 24 to 45 years (median 34), with 14 women being nulliparous. Six categories and 14 themes emerged from the analysis (Table 1). From the in-depth interviews, most women had more than one reason that convinced them of their decision.

**Table 1:** Main categories and themes of maternal reasons for cesarean birth

Category	Themes
I. Fear of childbirth	- Fear of labor pain
	- Fear of facing two painful events (failure of vaginal delivery and cesarean section)
	- Fear of harming the baby
II. Safety concerns related to health risk perceptions	- Underlying medical diseases (diabetes mellitus, heart diseases and HIV)
	- Biological risks (advanced maternal age and obesity)
	- Reproductive health problem (infertility)
III. Previous negative birth experiences	- Inadequate pain control
	- Dystocia
	- Baby injury
IV. Positive attitudes toward cesarean delivery	- Advantages of cesarean delivery
	- Disadvantages of vaginal delivery
V. Access to biased information	- Personal advice
	- Mass media
VI. Superstitious belief in auspicious birth dates	- Good fortune

### Category 1: fear of childbirth

Fear of childbirth was the main concern, even though seven women never had experienced childbirth before. They had fears for labor pain and facing two painful events: 1. failing vaginal birth and then; 2. having to undergo emergency cesarean section as well as fear of harming the baby.

*“I have a fear of pain during labor and delivery. I have heard that it is the most severe pain in one’s life.”*

*“I have a fear of labor pain, even though I had two spontaneous vaginal deliveries, and this was my third time.”*

### Category II: safety concerns related to health risk perceptions

Seven women perceived that they had significant risk factors potentially leading to unsafe vaginal birth as well as fetal jeopardy. These included underlying medical diseases (such as heart disease, diabetes mellitus and HIV), biological risks (advanced age and obesity), infertility and even the perception of a big baby as a consequence of maternal diabetes mellitus. They believed that they were not healthy and might not have enough power to push the baby out, thus resulting in injury to the baby.

*“I have an underlying heart disease, I am experiencing dyspnea sometimes, so I am afraid that I cannot push the baby, or may have worsening dyspnea during pushing, and my baby will be obstructed for a long period and be unsafe.”*

*“I am getting older, and have diabetes mellitus with a history of preeclampsia from a previous pregnancy. I am afraid that I have not enough power to push.”*

*“I have an HIV infection. I am afraid that my baby might have contact with blood or mucous in my vagina during delivery. I prefer cesarean delivery because I have heard that it reduces the risk of infection to my baby.”* (She had received standard antiretroviral drugs)

Two women had multiple risk factors: medical diseases, advanced age, infertility and maternal perception of a big baby, leading to the preference of cesarean birth.

*“I have been married for 6 years, and never got pregnant. Now, I am having my first pregnancy by IVF, after 3 attempts. I am now 45 years old, and also have diabetes mellitus arising during pregnancy, furthermore I am on diet control as well as insulin injections to control blood sugar. I fear that my baby is big at the time of delivery, so I am afraid that my baby will not be safe during vaginal delivery.”*

### Category III: previous negative birth experiences

Previous negative birth experiences also had a strong impact on pregnant women. Traumatic birth leads to fear of giving birth in subsequent pregnancies, as they perceived childbirth harmful for themselves and their babies. Inadequate analgesia during labor is also a problem, resulting in fear of pain during birth.

*“My first baby was delivered by vacuum extraction with much difficulty. His head was swelling, though it recovered a few days later. I don’t want to have this problem again.”*

*“I have had a bad experience of my previous pregnancy. I delivered with much difficulty, having labor pain for 2 days and was referred to the provincial hospital for cesarean section. However, cesarean section was not performed as expected. I was given drugs to enhance uterine contractions for such a long period with terrible pain, though ended up with successful vaginal delivery.”*

### Category IV: positive attitudes toward cesarean birth

Many women had positive attitudes toward cesarean birth, appreciating advantages over vaginal birth in terms of convenience, short delivery time without labor pain and so forth. Four women thought that cesarean section was a safe procedure, without terrible pain as compared to vaginal birth, as well as with less blood loss. Four women thought that it was worth to choose cesarean birth for tubal ligation. Women with fibroids, sometimes also wished to have these removed during cesarean section.

*“I feel that cesarean birth is safe for both mother and baby. Blood loss is less than vaginal birth.”*

*“Cesarean birth is faster than vaginal birth.”*

*“Planned cesarean sections are convenient, date and time can be selected, and there is no need to wait for spontaneous labor.*

*“I have a tumor in the womb, and I would like to have it removed at the time of cesarean section.”*

### Category V: access to biased information

Women have obtained information from either personal advice or mass media. Personal resources including words, experiences or advice from people, such as friends or relatives about their negative or positive birth experiences, may have had an influence on their decisions.

*“My relative struggled with shoulder dystocia. I am afraid that I may have the same problem, so I would like to have cesarean birth.”*

*“My friend told me that she had cesarean section with a spinal block, and it was not painful.”*

Currently, people can access various sources of information very easily, including social media, such as Facebook, wherein anybody can share experiences, ideas or comments, and this may have an influence on women’s decisions. Information from television, newspapers or movies may also present some information leading to misunderstanding about the real concepts and factors with regard to mode of birth.

*“I have read comments shared on Facebook and the internet, saying that vaginal birth is very painful.”*

*“I have searched for information about cesarean section, it is an alternative route of delivery with a short, painful period.”*

### Category VI: superstitious belief in auspicious birth dates

Three women believed in destiny. If their babies could be born on an auspicious date and time, they will be prosperous. This is a strong ideology in some families and cesarean birth has an advantage due to this issue.

*“I prefer cesarean birth because I can set an auspicious time for my baby.*”

## Discussion

Several reasons were revealed by women who preferred cesarean birth. Fear of childbirth, safety concerns related to health risk perceptions, previous negative birth experiences, positive attitudes toward cesarean birth, access to biased information and superstitious beliefs in auspicious birth dates were found in this study.

Almost all aspects were in accordance with previous studies, except for the personal superstitious belief in auspicious birth dates, which has been found only in some Asian countries (such as China, India, Taiwan and Thailand) [10–13, 15]. Having fibroids removed during cesarean section as an advantage was mentioned by some participants. This, however, was a misunderstanding, because this procedure may cause profound or uncontrolled hemorrhage, which may lead to hysterectomy.

Fear of childbirth was the most common reason for cesarean birth preference, which was similar to previous studies [10–13, 15, 16]. Obstetricians should explore the fear, because fear of pain cannot be managed by cesarean section, which by itself will lead to pain afterwards. Providing proper recommendations for women with fear of childbirth, such as analgesia for pain relief, risks and benefits of vaginal and cesarean birth, during antenatal care is essential. Safety concerns related to health risk perceptions were also common in this study. Our institution is a referral center in the South of Thailand and thus has a high proportion of complicated pregnancies. Due to this issue, obstetricians should clarify any misunderstandings, misbeliefs, or misperception, and the exact risks and benefits with regard to modes of birth, as serious complications may follow cesarean birth: especially abnormal placental adherence in subsequent pregnancies. Safety concerns based on health risk perceptions have also been mentioned in previous studies [10, 13].

Previous studies reported that negative birth experiences have been recognized as a strong factor for cesarean preference [10–12, 17]. History of traumatic births made women fear birth, and hence the request for planned cesarean section in order to avoid such bad events. In our study, women expressed their feelings on how they suffered from birth experiences in three aspects, including inadequate pain control, dystocia and injury to the baby. This reflects quality of care during labor, as pain control should be offered in addition to mental support. Dystocia is associated with severe pain [18]. Traumatic birthing makes women stressful, frustrated and depressed [19]. As no one wants her baby to be injured, these events can have a strong impact on cesarean birth preference.

One important factor is related to positive attitudes toward cesarean birth, which may be related to lack of health literacy. Women appreciated the advantages of cesarean birth in terms of convenience, short delivery time and less pain. Some women believed that the procedure to be safe and lacked knowledge with regard to serious complications in subsequent pregnancies. Knowledge has an influence on attitudes and with correct knowledge attitudes might change [20]. Most women who had positive attitudes toward uncomplicated vaginal birth would prefer that in their present pregnancies [21].

Information from mass media could dictate one’s ideas or beliefs [15]. Perceptions and interpretation were different among people based on their experiences, beliefs, critical thinking and reasoning. Because of one-way communication, misleading information can occur. Obstetricians should therefore clarify misbeliefs or misunderstandings for pregnant women. If women could get informative, professional and correct information, they may change their attitudes toward mode of birth, which may lead to decreasing rates of cesarean birth on demand.

Finally, a striking reason in our study was superstitious beliefs in auspicious birth dates. To our knowledge, this cultural preference is not found in most western populations, although superstitious beliefs in auspicious dates and times of birth is quite common in Thai society, as well as Chinese families. A study in California found a large number of Chinese births on the auspicious dates of the 8th, 18th and 28th day of the month, but no corresponding increase among Whites [22]. In Chinese culture, “8” is the luckiest number due to its association with wealth and luck. “Eight” in Chinese is pronounced “ba” and sounds similar to “fa” (fa cai), meaning wealth or fortune. Some people have strong beliefs in destiny, wherein birth time determines the course of their life. If they were born during an unlucky period, they would have bad luck throughout life, coupled with beliefs in astrology. Parents desire to provide the best opportunity for their children, so if they can choose an auspicious time to give birth, they would do so. It is a challenge to deal with personal beliefs and ideology, besides providing information about adverse consequences of elective cesarean birth for mothers and babies. Obstetricians could simply decline to perform cesarean section on maternal request, but this obviously may lead to conflict. It would possibly make the woman and her family choosing another obstetrician who would be willing to perform surgery, especially in the private sector.

Our qualitative study contributed data from pregnant Thai women, reflecting cultural preferences in Thailand with a different culture from that of western countries. It is a challenge for obstetricians to approach, since such ideas and belief systems are difficult to change. Such beliefs may not be related to education level, whereas family backgrounds seem to be more influential.

Strength of this study was that two investigators performed the content analysis independently and then discussed results to obtain consensus. This increases study validity, because it is obvious that different researchers may have disparate conclusions from the same data. The two investigators have different professional backgrounds (nurse and obstetrician), so that our findings were approached and discussed from different angles to ensure consistency in the analysis.

Previous studies have reported reasons which were not found in our study, such as fear of pelvic floor injury and urinary problems or encouragement from health personnel [13, 15]. Since cesarean section on maternal request is not allowed in our institution, some women who preferred cesarean birth and were aware of our hospital policy, may have gone to the private sector instead. Obstetricians’ preference and hospital policies might have more influences on women’s decisions. Some doctors prefer cesarean to vaginal birth because it is faster, more convenient and more profitable [8]. Limitation of our study was that it did not represent pregnant women in the private sector where cesarean birth on demand is permitted. In addition, in the context of very high cesarean rates in our institution, it may have an influence on pregnant women’s and doctors’ views toward cesarean birth.

## Conclusion

Many reasons for cesarean birth emerged from pregnant Thai women. One striking reason was superstitious beliefs in auspicious birth dates, which was a challenge for obstetricians to approach. Obstetricians should explore in detail why women request cesarean birth and provide effective counseling in order to decrease cesarean birth on demand.

## Supplementary Information

**Additional file 1.** Interview guide for in-depth interviews.

## Data Availability

The datasets generated and/or analysed during the current study are in Thai language and are not publicly available due to confidentiality of the participants, but are available from the corresponding author on reasonable request.
