# Supplementary material for: Why do pregnant women prefer cesarean birth? A qualitative study in a tertiary care center in Southern Thailand
Source: BMC Pregnancy Childbirth. 2021 Jan 6;21:23. doi: 10.1186/s12884-020-03525-3 (PMC7789505; doi:10.1186/s12884-020-03525-3)
Supplement: Supplementary file 1 — Additional file 1. Interview guide for in-depth interviews. [file 12884_2020_3525_MOESM1_ESM.docx]

**Interview guide for in-depth interviews**

| **Questions** | **Probes** |
| --- | --- |
| Could you please give me some personal information: such as age, occupation, income, marital status, education level, number of children, and so forth? |  |
| How do you feel about your current pregnancy? |  |
| What, if you have, is your birthing plan? | - Where do you plan to give birth? - Why did you choose that place? - Which kind of service care do you prefer, private or public? and why did you choose this? |
| Where do you find information about childbirth? | - If she has sought information by herself,   - From people, who are they?   - From mass media, what sources? |
| Which mode of birth do you prefer for this pregnancy? | - Why do you prefer to have cesarean birth? - Why didn’t you choose vaginal birth? - What do you think about vaginal birth? - Did you consult anyone regarding mode of birth? Who? |
| Could you please tell me the reasons why you choose to have cesarean birth? | - If fear, what do you fear? Why do you think like that?   - Labor pain   - Injury to your baby   - Injury to yourself   - Anything else? - If any health risk concerns, could you please tell me about them?   - Your disease, complication or any risk?   - What do you expect about the consequences of your disease/complication or any risk on childbirth, baby or yourself? - If previous negative birth experience, could you please tell me about it? - You said this……..could you explain this point further? |
| Who has influenced you to choose cesarean birth? | - Who is the most influential person? - What information you have received from influential people? - What did they say about cesarean birth? |
| What is/are the benefit(s) of cesarean birth? | - Which advantages of cesarean birth have an influence on your decision making? - You said this….…could you please explain this point further? |
| Is there anything else you would like to tell me? |  |
